# Clonal Selection of a Novel Deleterious TP53 Somatic Mutation Discovered in ctDNA of a KIT/PDGFRA Wild-Type Gastrointestinal Stromal Tumor Resistant to Imatinib

**DOI:** 10.3389/fphar.2020.00036

**Published:** 2020-02-07

**Authors:** Chiara Dalle Fratte, Michela Guardascione, Elena De Mattia, Eugenio Borsatti, Roberta Boschetto, Angelo Farruggio, Vincenzo Canzonieri, Loredana Romanato, Rachele Borsatti, Sara Gagno, Elena Marangon, Maurizio Polano, Angela Buonadonna, Giuseppe Toffoli, Erika Cecchin

**Affiliations:** ^1^Experimental and Clinical Pharmacology Unit, Centro di Riferimento Oncologico di Aviano (CRO) IRCCS, Aviano, Italy; ^2^Nuclear Medicine Unit, Centro di Riferimento Oncologico di Aviano (CRO) IRCCS, Aviano, Italy; ^3^Pathology Unit, ULSS 17 Este-Monselice, Este, Italy; ^4^Department of Medical, Surgical and Health Sciences, University of Trieste, Trieste, Italy; ^5^Pathology Unit, Centro di Riferimento Oncologico di Aviano (CRO) IRCCS, Aviano, Italy; ^6^Medical Oncology Unit, Centro di Riferimento Oncologico di Aviano (CRO) IRCCS, Aviano, Italy

**Keywords:** circulating tumor DNA, *TP53*, gastrointestinal stromal tumor, imatinib, liquid biopsy

## Abstract

The standard of care for the first-line treatment of advanced gastrointestinal stromal tumor (GIST) is represented by imatinib, which is given daily at a standard dosage until tumor progression. Resistance to imatinib commonly occurs through the clonal selection of genetic mutations in the tumor DNA, and an increase in imatinib dosage was demonstrated to be efficacious to overcome imatinib resistance. Wild-type GISTs, which do not display KIT or platelet-derived growth factor receptor alpha (PDGFRA) mutations, are usually primarily insensitive to imatinib and tend to rapidly relapse in course of treatment. Here we report the case of a 53-year-old male patient with gastric GIST who primarily did not respond to imatinib and that, despite the administration of an increased imatinib dose, led to patient death. By using a deep next-generation sequencing barcode-aware approach, we analyzed a panel of actionable cancer-related genes in the patient cfDNA to investigate somatic changes responsible for imatinib resistance. We identified, in two serial circulating tumor DNA (ctDNA) samples, a sharp increase in the allele frequency of a never described TP53 mutation (c.560-7_560-2delCTCTTAinsT) located in a splice acceptor site and responsible for a protein loss of function. The same TP53 mutation was retrospectively identified in the primary tumor by digital droplet PCR at a subclonal frequency (0.1%). The mutation was detected at a very high allelic frequency (99%) in the metastatic hepatic lesion, suggesting a rapid clonal selection of the mutation during tumor progression. Imatinib plasma concentration at steady state was above the threshold of 760 ng/ml reported in the literature for the minimum efficacious concentration. The *de novo* TP53 (c.560-7_560-2delCTCTTAinsT) mutation was *in silico* predicted to be associated with an aberrant RNA splicing and with an aggressive phenotype which might have contributed to a rapid disease spread despite the administration of an increased imatinib dosage. This result underlies the need of a better investigation upon the role of TP53 in the pathogenesis of GISTs and sustains the use of next-generation sequencing (NGS) in cfDNA for the identification of novel genetic markers in wild-type GISTs.

## Background

Gastrointestinal stromal tumors (GISTs) are the most common soft tissue tumors arising in the gastrointestinal tract. Common sites of GIST in the gastrointestinal tract include stomach (50%), small intestine (25%), rectum (5%), esophagus (< 5%), while extra-intestinal localizations are rare (< 5%). (DeMatteo et al., 2000; Casali et al., 2018) The diagnosis of GIST commonly relies on immunohistochemical (IHC) analysis of tumor tissue and is based on the assessment of KIT and DOG1 positivity. Based on histopathological features including mitotic index, tumor size, and primary site, risk-stratification schemes have been formulated. (Fletcher et al., 2002; Miettinen and Lasota, 2006).

By a molecular point of view, GISTs are characterized by gain-of-function mutations in *KIT* (70%–75% of cases) or *PDGFRA* (platelet-derived growth factor receptor, alpha polypeptide) genes (5%–10% of cases). (Corless et al., 2004) The mutational status of *KIT/PDGFRA* represents a significant predictive factor for response to the targeted drug imatinib mesylate. In particular, patients displaying *KIT* exon 11 mutations are usually sensitive toward imatinib, whereas patients bearing *KIT* exon 9 mutations are less sensitive and benefit from a higher drug's starting dosage. (Debiec-Rychter et al., 2006) On the other hand, *KIT* exon 13 and 17 mutations are usually insensitive to imatinib and commonly arise later during treatment, leading to secondary acquired resistance. (Lasota et al., 2008) Concerning the less common *PDGFRA* mutations, the p.D842V substitution is associated to primary insensitivity to imatinib, thus suggesting an alternative drug, i.e., sunitinib. (Heinrich et al., 2003) The subpopulation of GIST patients who do not show *KIT* or *PDGFRA* mutations has been historically classified as “wild-type” and only in the last years the contribution of other genes, such as *BRAF*, *SDH*, and *NF1*, has emerged to play a role in the pathogenesis of GIST (Boikos et al., 2016).

Only a narrow panel of mutations is known to be directly associated with the primary or secondary resistance to imatinib. Indeed, so far, imatinib resistance is mainly attributed to mutations located in *KIT*, *PDGFRA, BRAF*, and *SDH* genes, and thus other “noncanonical” genes remain less investigated. The inclusion of other oncogenes into the panels which are routinely screened for imatinib treatment monitoring could lead to the identification of novel biomarkers for the early diagnosis of treatment failure. Moreover, the use of wider panels of genes could be of particular interest in *wild-type* GIST, for which the dynamic tracking of driver known mutations is not feasible.

The strategy of tumor mutations dynamic monitoring for early detection of imatinib resistant clones is of special worth, as alternative therapeutic approaches, after imatinib failure, are available in the clinical care, such as sunitinib and regorafenib. A suitable method for the dynamic monitoring of tumor behavior is offered by the circulating tumor DNA (ctDNA) analysis. In the framework of liquid biopsies, ctDNA represents a specific biomarker which displays the same molecular characteristics (i.e., mutations) of the tissue of origin thus representing a real-time source of tumor-derived DNA. In the clinical setting, ctDNA analysis has shown to be of prognostic significance in predicting the targeted therapy's response, as well as in the early identification of disease relapse and/or progression (Tie et al., 2016; Coombes et al., 2019; Siravegna et al., 2019).

With the aim of developing an innovative approach for the dynamic monitoring of imatinib in GIST patients, we set up a joint research project for the ctDNA analysis and for the monitoring of imatinib plasma C_min_ in blood samples collected during patients' follow-up. The ctDNA analysis, by means of targeted deep sequencing, is focused on detecting tumor-related mutations which could be informative about disease status and treatment response.

## Case Presentation

**Figure 1:**
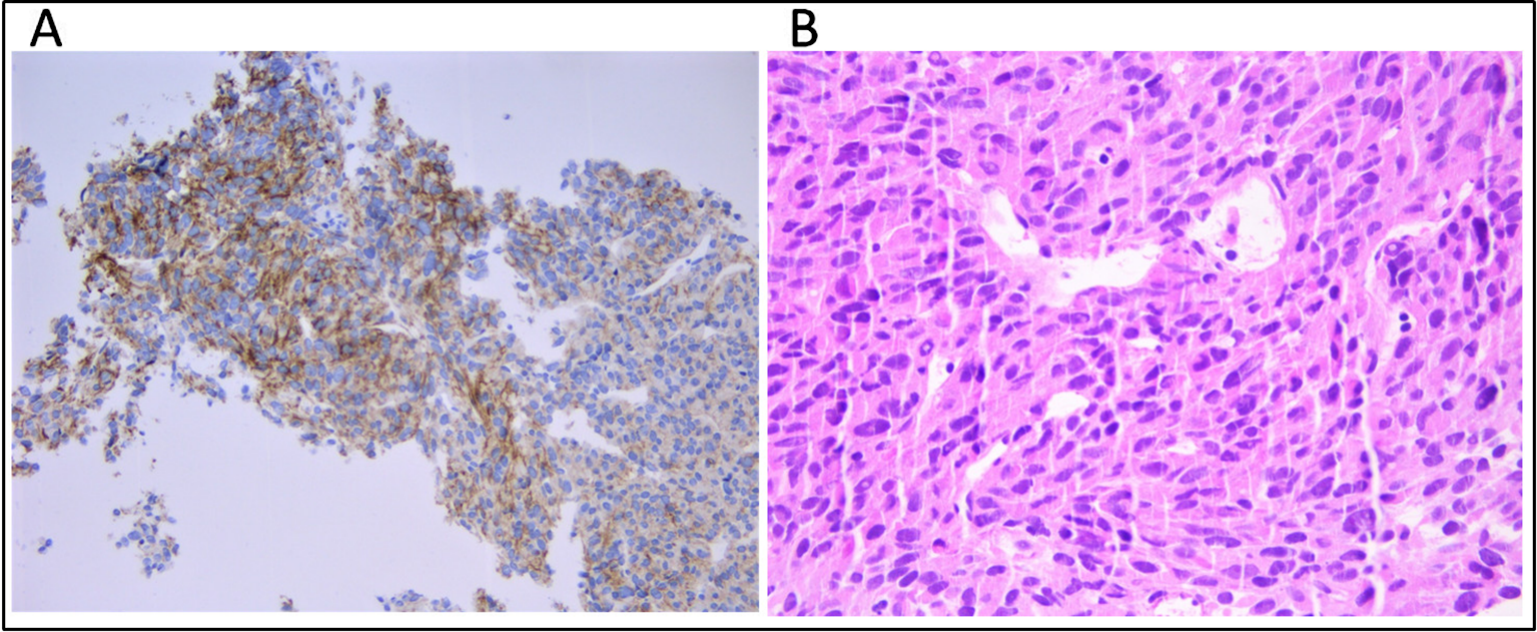
**(A)** Immunhistochemical staining for CD117 (c-KIT) and **(B)** tumor composition of spindle cells and eosinophilic cytoplasm (hematoxylin and eosin) on the metastatic hepatic tissue.

A 53-year-old male patient was diagnosed with gastric GIST in May 2015 at Azienda Ospedaliera ULSS 9 of Monselice (PD) for which he underwent a total gastrectomy with no evidence of residual disease. The tumor tissue examination revealed the characteristic spindle cell morphology of GIST and displayed a low mitotic index (< 1/50 HPF). Immunohistochemical stain revealed positivity for Ki67 and CD117 (c-KIT) antigens, confirming the diagnosis of GIST, whereas stains for smooth muscle alpha-actin, desmin, CD34, and S-100 were negative. A *post hoc* molecular analysis did not highlight any somatic mutation in *KIT* or *PDGFRA*, allowing to define it as a wild-type tumor. The patient was classified as a low risk of recurrence, and the wait-and-see approach was preferred to adjuvant treatment with imatinib. In November 2015, magnetic resonance showed the presence of six hepatic nodules with maximum diameter of 2.5 cm consistent with metastatic GIST lesions, so imatinib first-line therapy was started at the standard dosage of 400 mg/day. In March 2016, the patient accessed medical care in our hospital in which a magnetic resonance showed hepatic disease progression. The GIST derivation of hepatic lesions was confirmed through a tissue biopsy staining positively for c-KIT antigen (**Figure 1A**); therefore, imatinib dosage was increased to 800 mg/day. In October 2017, PET imaging revealed further hepatic disease progression in addition to bone and intra-abdominal metastatic spread, so the patient was switched to sunitinib. During the overall course of therapy, the patient displayed a primary resistance against imatinib since he never experienced a clinical benefit from treatment. The patient died because of disease progression in March 2018.

## Methods

(For a detailed description of *Methods*, see **Supplementary Methods**).

### Biological Sample Collection and Ethics Approval

The patient provided a signed informed consent at the time of enrollment. Blood samples were collected in January 2017 (sample IM_21.1) and in July 2017 (sample IM_21.2). A diagnostic residue of the formalin-fixed paraffin-embedded (FFPE) bioptic tissue derived from the hepatic lesion was provided by the Pathological Anatomy Division of IRCCS CRO, whereas primary FFPE surgical tissue was kindly provided by Azienda Ospedaliera ULSS 9 of Monselice (PD).

### DNA Extraction and Quality Control (QC)

Cell-free DNA (cfDNA) was extracted from 4 ml of plasma using the QIAamp MinElute ccfDNA Kit (Qiagen) and quantified with Quantus Fluorometer (Promega). Fragment size distribution was assessed by High Sensitivity TapeStation (Applied Biosystems). Germline DNA was extracted from 200 μl of buffy-coat using the automated BioRobot EZ1 (Qiagen). DNA from FFPE tissue (both primary tumor and hepatic metastasis) was extracted using the GeneRead DNA FFPE Kit (Qiagen) according to the manufacturer's instructions. The median tumor cell content was 80%, as established by a trained expert pathologist (**Figure 1B**).

### Library Preparation, Sequencing, and Data Analysis

Genomic libraries were prepared using QiaSeq Human Actionable Solid Tumor Panel DNA (Qiagen). Regions covered by the panel are listed in **Supplementary Table S1**. Pooled libraries were paired-end (151 × 2) sequenced in an Illumina platform (MiSeq). Bioinformatic analysis was performed on a workstation with a 30-core Intel Core i7 and 64 GB of memory running Centos 7.5. Raw reads after trimming for quality were aligned against the reference genome hg19 (UCSC) using bwa aligner. (Li and Durbin, 2009) Variants were called using smCounter v 2 with default parameters. (Xu et al., 2019) Identified variants were manually verified using Integrative Genomics Viewer (IGV, https://www.broadinstitute.org/igv).

### ddPCR Assay

cfDNA, DNA from primary tumor, and DNA from metastatic tissue were interrogated for the presence of *TP53* indel (c.560-7_560-2delCTCTTAinsT) by a ddPCR custom assay developed from BioRad (Bio-Rad Laboratories, Hercules, CA, USA). ddPCR was performed using primers and specific TaqMan probes targeting the wild-type *TP53* sequence and the aberrant *TP53* sequence bearing the indel c.560-7_560-2delCTCTTAinsT. As reference mutated control, a synthetic oligonucleotide bearing the *TP53* indel c.560-7_560-2delCTCTTAinsT was used (Sigma Aldrich, St. Louis, MO, USA). As a reference wild-type control, a germline DNA in which the presence of the *TP53* indel (c.560-7_560-2delCTCTTAinsT) was excluded through next-generation sequencing (NGS) was used. Droplet generation was performed using QX200™ Droplet Generator™ (Bio-Rad Laboratories, Hercules, CA, USA), and fluorescence emitted from droplets was measured using QX200™ Droplet Reader (Bio-Rad Laboratories, Hercules, CA, USA). Sample analysis was performed using QuantaSoft v1.7.4.0917 software (Bio-Rad Laboratories, Hercules, CA, USA). Details concerning ddPCR conditions and data analysis are reported in **Supplementary Methods**.

### Computational Prediction of Splicing Alteration

Six freely available *in silico* tools were used to predict the impact of the splice-site mutation in *TP53* gene on pre-mRNA splicing. Tools used are SpliceView (http://bioinfo.itb.cnr.it/~webgene/wwwspliceview.html), GENSCAN (http://hollywood.mit.edu/GENSCAN.html), NetGene2 (http://www.cbs.dtu.dk/services/NetGene2/), MaxEntScan (http://hollywood.mit.edu/burgelab/maxent/Xmaxentscan_scoreseq.html), and Human Splicing Finder (HSF, http://www.umd.be/HSF/HSF.shtml) (Shapiro and Senapathy, 1987; Brunak et al., 1991; Hebsgaard et al., 1996; Burge and Karlin, 1997; Reese et al., 1997; Yeo and Burge, 2004; Houdayer et al., 2008; Desmet et al., 2009). To facilitate the output interpretation, we compared the score of the mutant with the score of the reference sequence.

### Liquid Chromatography Tandem Mass Spectrometry (LC-MS/MS) Quantification of Imatinib Plasma Concentrations

The quantification of imatinib was obtained using an LC-MS/MS apparatus consisting of a Prominence UFLC XR (Shimadzu) coupled with an API 4000 QTrap mass spectrometer (SCIEX). Details concerning sample processing and experimental conditions are reported in **Supplementary Methods**.

## Results

### Next-Generation Sequencing of DNA Derived From Plasma (cfDNA), Primary Tumor Tissue, and Metastatic Tissue

After variants calling by smCounter v2, genetic variants were filtered *per* the following criteria: passing filter (PASS), quality score ≥100, frequency of mutated allele ≥0.5%, and total number of reads mapping the chromosomal location (reads depth) ≥2,500 X. All the genetic variants were compared to those obtained by matched germline DNA sequencing to exclude from the analysis nonsomatic variants.

In the two serial cfDNA samples, one somatic indel affecting the exon 6 flanking site of *TP53* gene indel (c.560-7_560-2delCTCTTAinsT) at nucleotide position c.560-2_c.560-7 was identified at a minor allele frequency (MAF) of 2.7% in the first plasma sample (IM_21.1) and of 9.7% in the second plasma sample (IM_21.2). No other somatic mutation was detected in cfDNA. The same *TP53* indel was detected through NGS in the DNA from the metastatic tissue with a MAF of 99%. The mutation identified is an intronic indel that affects the canonical AG/GT splice site motif by the deletion of the nucleotide in position –2 upstream exon 6.

The identified variant was automatically annotated against the human *TP53* genomic sequence NC_000017.10 (chr 17:7,571,720-7,590,868) corresponding to isoform NM_00546.5. The latest release of the International Agency for Research on Cancer (IARC) *TP53* Mutation Database (Database R19, released on August 2018) (http://p53.iarc.fr/TP53GeneVariations.aspx) was used to check the *de novo TP53* variant, confirming our novel finding. (Bouaoun et al., 2016) The characteristics of *TP53* mutation are listed in **Table 1**.

**Table 1:** *TP53* somatic mutation identified by next-generation sequencing (NGS). Genomic coordinates of the mutation and the read depth in that chromosomal location are reported.

Sample ID	Genomic coordinates	Read depth	UMT	VMF	Mutation frequency (%)	cDNA change	Type of mutation
**IM_21.1**	17:7,578,291	6,672	1,023	28	2.7	c.560-7_560-2delCTCTTAinsT	Indel
**IM_21.2**	17:7,578,291	5,868	876	85	9.7	c.560-7_560-2delCTCTTAinsT	Indel

The total number of reads bearing the same Unique Molecular Target (UMT) and those reporting the mutation [Variant Mutational Fraction (VMF)] was used to calculate the mutation frequency in each sample.

DNA derived from the primary tumor tissue at surgery resulted wild-type for the regions analyzed, as no somatic mutation was detected.

### ddPCR Analysis on Plasma, Primary Tumor Tissue, and Metastatic Tissue-Derived DNA

The custom ddPCR assay was harnessed to validate the presence of *TP53* indel (c.560-7_560-2delCTCTTAinsT) in cfDNA and metastatic tissue. Primary tumor tissue was interrogated as well to perform a cross-platform comparison and increase the sensitivity in mutation detection.

**Figure 2:**
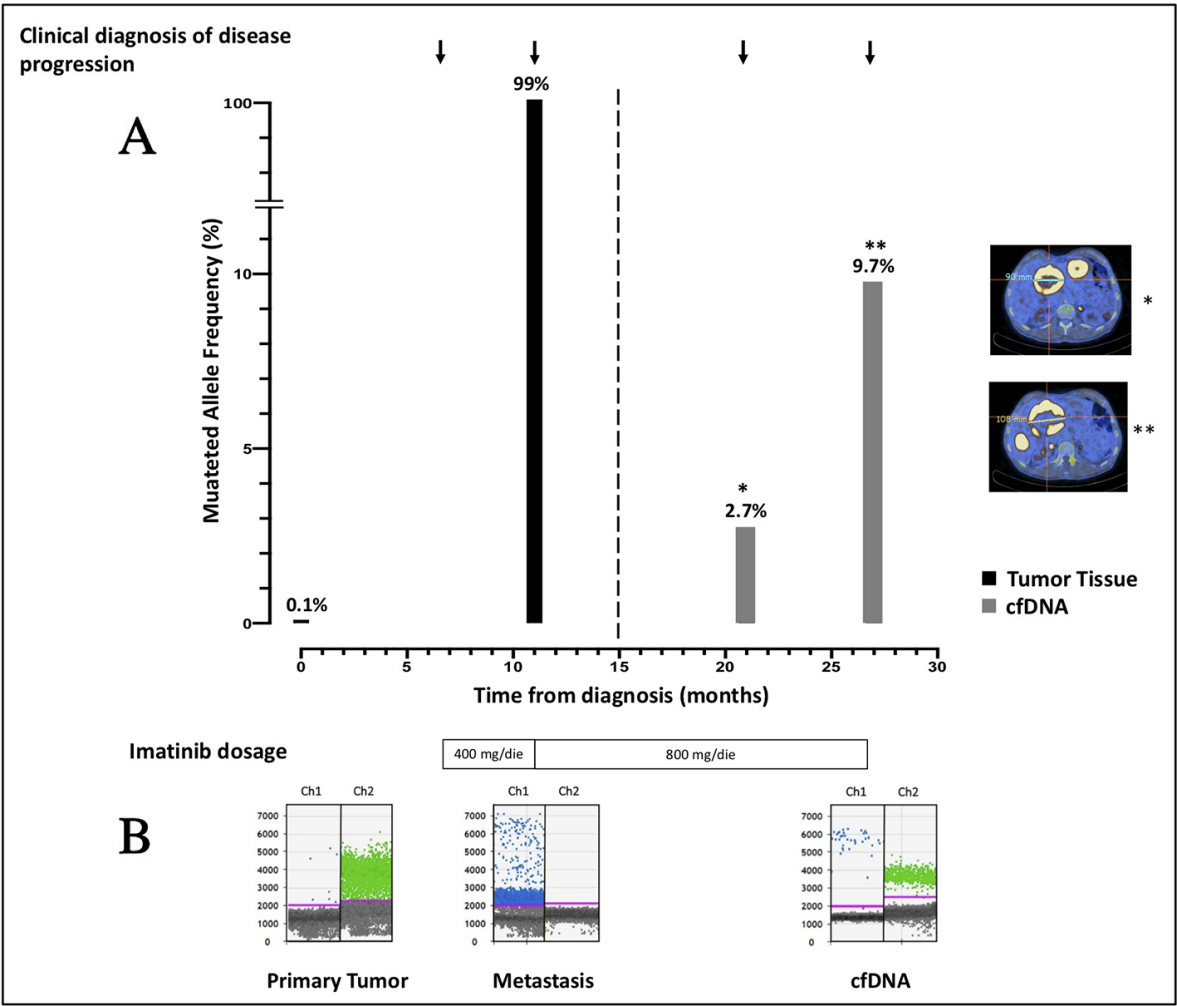
**(A)** Course of disease from the time of diagnosis, treatment administered, PET/CT images, and allele frequency of *TP53* indel are shown. The mutated allele frequency in tumor tissue (black columns) is reported for the primary tumor [minor allele frequency (MAF) 0.1%, ddPCR] and for the metastatic lesion (MAF 99%, ddPCR) at the time of surgery and biopsic sampling, respectively. The ctDNA fraction (gray columns) is reported for the sample IM_21.1 [MAF 2.7%, next-generation sequencing (NGS)] and sample IM_21.2 (MAF 9.7%, NGS). PET/CT scans reporting the diameter of target lesions and performed in concomitance to blood sampling are shown as well. On the bottom of the plot, the imatinib dosage administered is indicated. **(B)** ddPCR plots reporting the signal generated from the wild-type (green dots) and the mutated (blue dots) sequence are shown. In chronological order are reported the primary tumor DNA, the metastatic DNA, and the IM_21.1 cfDNA.

Since cfDNA IM_21.1 was completely depleted to perform NGS, only cfDNA IM_21.2 was analyzed through ddPCR. In cfDNA IM_21.2, the presence of the *TP53* indels was confirmed by ddPCR, which revealed 16 mutated copies/µl, corresponding to 277 mutated copies/ml of plasma. So, in cfDNA, the MAF estimated by ddPCR was 17%, superior to that reported by NGS (9.7%). The MAF revealed in the metastatic tissue-derived DNA by ddPCR was comparable to that obtained by NGS analysis, confirming a complete selection of the mutated clone in the metastatic lesion (MAF ~100%). Notably, the use of ddPCR allowed the identification of *TP53* indel also in the DNA derived from the primary tumor tissue with a mutated allele frequency of 0.1% that was not detectable by means of NGS (**Figure 2**).

### Computational Prediction of *TP53*c.560-7_560-2delCTCTTAinsT

**Figure 3:**
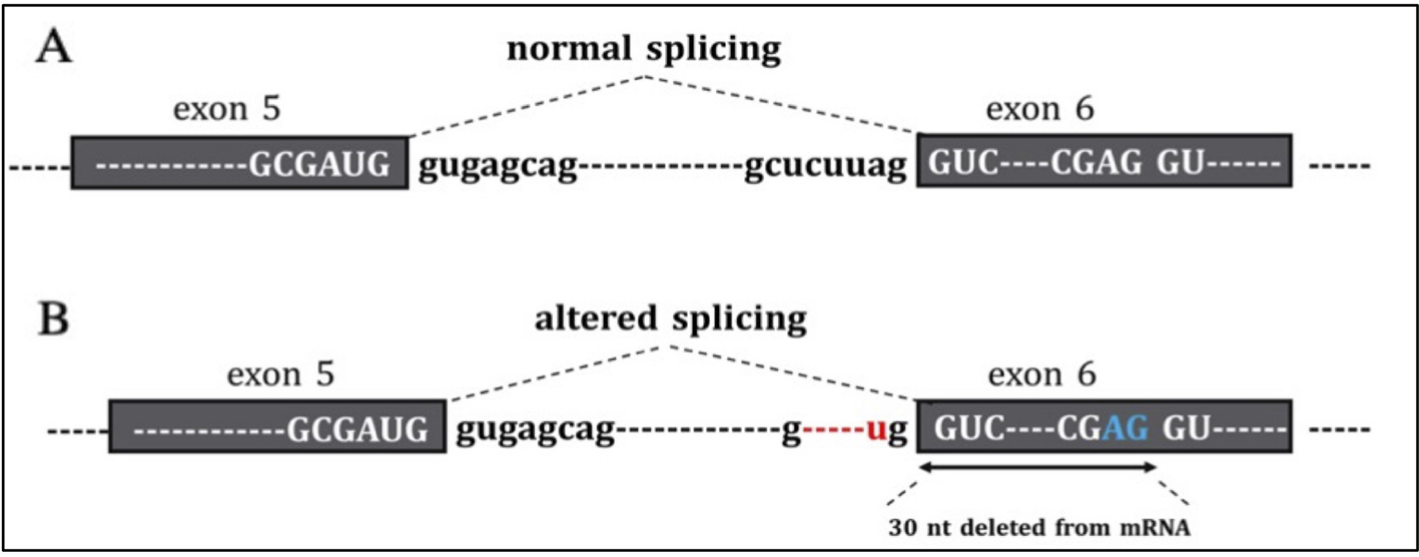
In the figure are reported **(A)** the normal sequence and exon splicing of TP53 pre-mRNA (exons 5–6) and **(B)** aberrant splicing caused by the c.560-7_560-2delCTCTTAinsT (red) likely to generate an in-frame deletion of 30 nucleotides from mRNA due to the activation of a cryptic splice site (blu).

Tools used to predict the effect of the *TP53* indel at mRNA and the respective scores generated are listed in **Table 2**. All but one tool agree in identifying the canonical splice site in the wild-type *TP53* sequence, and all of them predicted the splice site destruction in the mutated sequence. Consequently, the effect of the mutation was supposed to be deleterious by five out of six predictive tools, not being evaluable by means of NetGene2, which did not detect the wild-type splice site. The activation of an alternative splice site was predicted by HSF that identified a likely new splicing acceptor site located 30 nucleotides downstream from the canonical site. The new splice site was scored 50.40 by HSF (**Table 2**), and it is weaker than the canonical ones, which was scored 80.49. The activation of the new cryptic splice site would lead to an in-frame deletion of 10 amino acids from the position 187 to 196 in the mature protein. The description of the *TP53* indel and its predicted effect on mRNA strand are depicted in **Figure 3**.

**Table 2:** Computational prediction of the effect of the mutation on the *TP53* splice site by the use of six different bioinformatic tools.

Tool	Output	Reference score	Mutated score	Predicted effect
**SpliceView**	Score (0–100)	83	Not detected	Deleterious
**GENSCAN**	Probability score (0–1)	0.120	Not detected	Deleterious
**NetGene2**	Confidence score	0.00	Not detected	Not evaluable
**NNSplice 0.9**	Score (0–1)	0.94	Not detected	Deleterious
**Human Splicing Finder** (**HSF**)	Score (0–100)	80.49	Not detected	Deleterious
**MaxEntScan**	Maximum entropy score	1.08	–2.91	Deleterious

The wild-type DNA sequence was compared with the mutated one, and the effect was predicted by comparing the two generated scores.

Molecular biology analyses to confirm the mutation's impact on TP53 mRNA splicing were attempted but not feasible due to the very poor quality of FFPE RNA (data not shown).

### Imatinib Plasma Levels

The sample IM_21.1 for C_min_ analysis was collected 17.5 h after the previous imatinib; drug plasma concentration was determined as 987 ng/ml. The sample IM_21.2 was collected a week after the last intake of the drug. Therefore, this last sample was not suitable for C_min_ determination given that the possible plasma imatinib concentration was not associated with steady state kinetics.

## Discussion

The use of imatinib has favorably rewritten the natural history of GIST, improving the patients' outcome in terms of survival. However, primary and secondary resistance is the main weakness of imatinib and represents the leading cause of progression.

The primary resistance is related to tumor's molecular features at baseline and is assessed on tumor at the time of diagnosis, as recommended by clinical guidelines. (Casali et al., 2018) Wild-type GISTs, characterized by the absence of *KIT*/*PDGFRA* activating mutations, are a heterogeneous class of tumors showing multiple genetic and morphological features. Therapeutic strategies for the treatment of these tumor subtypes are not defined yet.

Secondary imatinib resistance is commonly observed after 2 years of treatment in half of primarily responder patients as a consequence of the selective pressure exerted by the drug. In *KIT*/*PDGFRA*-mutated GISTs, the development of acquired resistance is commonly restricted to secondary mutations in these genes, enabling a more handling monitoring of disease progression by the detection of target mutations. In wild-type GISTs, which do not display a shared evolutionary path, the identification of genetic markers to assess tumor's evolution is urgently needed (Wei et al., 2018).

The possibility of interrogating ctDNA as a surrogate of tumor tissue by massive parallel sequencing has enabled the real-time detection of emergent resistance clones in several kinds of malignancies in a less invasive manner (Russo et al., 2016).

In this study, using a targeted NGS panel of hotspot regions of 23 cancer-related genes, we assessed the molecular evolution of a wild-type GIST by analyzing two serial cfDNA samples collected 6 months apart, the primary tumor tissue and the hepatic metastasis tissue. We found that a novel *TP53* indel (c.560-7_560-2delCTCTTAinsT) was detected in cfDNA samples with an allele frequency of 2.7% (IM_21.1) and 9.7% (IM_21.2). The primary and relapsed tumors did not show *KIT*/*PDGFRA* mutations, but they harbored the same *TP53* indel with an allele frequency of 0.1% and ~100%, respectively.

The functional impact of *TP53* indel (c.560-7_560-2delCTCTTAinsT) was postulated on the basis of its localization in a highly conserved region. We hypothesized a misrecognition of the canonical splice site from the splicing machinery, which would result in the translation of a truncated or nonfunctional protein. Our hypothesis was sustained by five different *in vitro* algorithms that predicted the canonical splice site destruction and the likely activation of noncanonical ones, located 30 base pairs downstream of the canonical one in the intron 5/exon 6 boundary. At the protein level, the TP53 region excised from canonical mRNA transcription, both in the case of a complete or a partial loss of exon 6, is of pivotal relevance for the interaction with other proteins involved in the cell cycle regulation, such as AXIN1, HIPK1, and ZNF385A and is located in the DNA-binding domain. (Das et al., 2007; Sun et al., 2009) The production of an aberrant *TP53* transcript leads to the transduction of a truncated and nonfunctional TP53 protein but could also drive the reduction of the TP53 expression as a consequence of a nonsense-mediated mRNA decay.

*TP53* is a tumor suppressor gene well known to play a pivotal role in the DNA repair process and in the apoptosis initiation. Its inactivation is a frequent event in cancer and is commonly associated with a worst prognosis (Basu and Murphy, 2016).

Although only few studies have investigated the role of *TP53* mutations in GIST, a consensus upon their association with imatinib resistance has been achieved. The first evidence demonstrating the correlation between *TP53* mutations and imatinib insensitivity was described by Wendel et al. (2006) in BCR-ABL-positive leukemic cells. They observed that the mechanism of imatinib resistance was independent of the chemical inhibition of BCR-ABL kinase by imatinib, suggesting a downstream involvement of *TP53* mutations in leading the drug's resistance. Further studies confirmed the loss of TP53 in chronic myeloid leukemia as a molecular feature associated with imatinib resistance (Al-achkar et al., 2012).

Recently, in a study aimed at identifying genes involved in imatinib resistance in GIST-T1 cells through a CRISP-Cas9 knockout genome-wide screening, Cao et al. (2018) identified *TP53* as one of the main genes associated to imatinib resistance. These evidences suggest that genomic alterations in genes related to the apoptosis pathway might represent an escape route exploited by tumor cells to evade imatinib therapy.

In *KIT*/*PDGFRA* mutant GISTs, there is no doubt upon the origin of the oncogenic signaling, and the development of imatinib resistance is mainly restricted to the acquisition of secondary *KIT*/*PDGFRA* mutant clones bearing novel mutations. In these groups of GISTs, the overall *TP53* mutation rate was reported as low, emphasizing the oncogenic reliance on kinase-mediated signaling. However, a straightforward association between presence of *TP53* aberrations and GIST malignancy has been observed, with a significant increase of *TP53* aberrations in high-risk rather than in low-risk tumors. (Merten et al., 2016; Ihle et al., 2018; Heinrich et al., 2019) On the other hand, *TP53* has emerged as one of the main mutated genes in wild-type GISTs, supporting its likely role in the pathogenesis of these tumor subtypes (Pantaleo et al., 2017).

In this clinical case study, the rapid metastatic evolution is consistent with the *TP53* mutant clonal selection from the primary to the relapsed tumor. The homozygous presence of *TP53* indel (c.560-7_560-2delCTCTTAinsT) in fundamentally all hepatic relapsed cells suggests once again an indisputable association between *TP53* deleterious mutations in GIST and the establishment of an aggressive phenotype insensitive to imatinib.

Moreover, the observation of no clinically actionable mutations, which might represent a molecular target for currently available therapeutic options, corroborates the lack of sensitivity toward imatinib reported here and implies the impossibility of prescribing further targeted drugs. Indeed, the administration of targeted therapies is limited to the presence of specific overexpressed or mutated molecular targets in tumor cells, thus making the management of wild-type tumors a challenging task.

In this case, the clinical tumor progression was well recapitulated by the longitudinal sequencing of ctDNA, which revealed the presence of *TP53* c.560-7_560-2delCTCTTAinsT at increasing allele frequency over 6 months. This finding is significant since it sustains the feasibility of relying on information obtained by liquid rather than tissue biopsies for the assessment of genetic features in metastatic GISTs. A good concordance between mutated cfDNA and tumor tissue in GIST patients was reported by previous studies, which observed a higher detection rate of ctDNA in patients with active disease and high tumor burden, rather than in patients with complete response or localized disease. (Maier et al., 2013; Xu et al., 2018) In this frame, the allele frequency of mutated cfDNA was shown to increase or decrease according to disease progression or tumor shrinkage, respectively, allowing the dynamic monitoring of tumor changes in advance GIST.

In sample IM_21.1, C_min_ resulted equal to 987 ng/ml (higher than the recommended threshold of 760 ng/ml); therefore, we reasonably consider adequate the imatinib level in patient's plasma and assume that his lack of response to therapy was not due to a concentration issue but more probably to the biological aggressiveness of the disease (Bouchet et al., 2016).

In summary, our work sustains the applicability of NGS of cfDNA for the monitoring of GIST patients on treatment with imatinib, and for the characterization of the mutational pattern of GISTs, especially in those classified as wild-type, for whom the identification of genetic markers is even more urgent due to the lack of targetable mutations. The screening of a panel of actionable genes offers the possibility of identifying new tumor markers, which may be relevant for the surveillance of tumor's evolution and for the development of new drugs. In the era of precision oncology, the baseline profiling of tumor is an imperative need for choosing the best therapeutic option and for avoiding the prolonged administration of ineffective drugs. Moreover, this procedure should be accompanied by the longitudinal follow-up of tumor genetics for the early identification of tumor changes and the emergence of resistance subclones. In this field, the use of liquid biopsy coupled with NGS represents a valuable tool to explore in parallel a wide range of genomic regions and to broader horizons upon tumor's evolutionary process. In the case here reported, the identification of a novel and deleterious *TP53* indel (c.560-7_560-2delCTCTTAinsT), compatible with an aggressive and drug resistance phenotype, remarks the need for further investigations upon the role of *TP53* in wild-type GISTs as well as on its involvement in the development of acquired resistance toward tyrosine kinase inhibitors. The clinical management of wild-type GIST remains a subject of open debate, and effective therapeutic strategies are still lacking. Even though this class of tumors usually displays an indolent course, the development of unpredicted outcomes such as the evolution into a more aggressive form must be considered. An accurate noninvasive molecular monitoring by the use of the liquid biopsy is of primary relevance to identify effective therapeutic strategies and to personalize the therapeutic strategies.

## Data Availability Statement

The data cannot be made publicly available because the consent was not acquired from the patient to publish his genetic profile. Reasonable requests for data should be directed to: ececchin@cro.it.

## Ethics Statement

The studies involving human participants were reviewed and approved by Comitato Etico Indipendente-Centro di Riferimento Oncologico di Aviano. The patients/participants provided their written informed consent to participate in this study. Written informed consent was obtained from the individual(s) for the publication of any potentially identifiable images or data included in this article.

## Author Contributions

CDF, EC, EDM, MG, and GT were involved in designing the study, critically revising the results, and preparing the manuscript, CDF, RBR, LR, MP, EM, and SG were involved in the molecular, bioinformatic and biochemical analyses. MG, AB, RBS and AF collected samples and clinical data, EB provided the PET/CT scans and VC did the histopathological analysis. All authors discussed the results and contributed to the final manuscript.

## Conflict of Interest

The authors declare that the research was conducted in the absence of any commercial or financial relationships that could be construed as a potential conflict of interest.
