# Supplementary material for: Clonal Selection of a Novel Deleterious TP53 Somatic Mutation Discovered in ctDNA of a KIT/PDGFRA Wild-Type Gastrointestinal Stromal Tumor Resistant to Imatinib
Source: Front Pharmacol. 2020 Feb 7;11:36. doi: 10.3389/fphar.2020.00036 (PMC7019050; doi:10.3389/fphar.2020.00036)
Supplement: Supplementary file 1 [file DataSheet_1.docx]

**Supplementary Methods:**

## Biological samples processing: Ten milliliters of blood were collected in K-EDTA tubes and centrifuged within 1 hour from drawn as follows: first centrifugation step was performed at 1,600 g for 10 min without brake and then the harvested surnatant plasma was centrifuged again at 3400 g for 10 min without brake to remove cell debris. Plasma was stored at -80 °C. The buffy coat was recovered after the first centrifugation step and stored at -80 °C too.

## Libraries preparation and QC: Libraries were prepared starting from 10 ng of cfDNA, 40 ng of germline DNA from buffy coat, or 250 ng of DNA from FFPE tissue samples, according to the manufacturer instructions. Briefly, DNA molecules were enzymatically fragmented, tagged with unique molecular indexes (UMI) and amplified in the targeted regions with multiplex PCR. Final libraries were quantified using Quantus Fluorometer (Promega) and fragment size distribution was assessed by using High Sensitivity Tapestation (Agilent Technologies).

## ddPCR: ddPCR reactions were set using 10 μL of ddPCR Supermix for probes (no dUTP), 1 μL of assay, 4 μL of PCR-grade water and 5 μL of DNA. All samples were analyzed in quintuplicate. As no template control (NTC) PCR grade water was used instead of DNA. Thermal cycling was performed as follows according to the manufacturer’s instructions: 95 ºC for 10 minutes (polymerase activation), 94 ºC for 30 seconds (denaturation), 56 ºC for 1 minute (annealing and elongation) and 98 ºC for 10 minutes (enzyme inactivation). Forty cycles of DNA amplification were carried out. Wild type and mutated copies were automatically calculated after manually removal of false-positive calls (*i.e.* droplets containing both templates) and was expressed as number of copies *per* µl loaded. Mutated allele frequency was manually calculated by dividing the number of droplets containing mutated copies for the number of positive droplets.

*In silico* tools: The backbone of all software relies on algorithms which predict the likely position of exon-intron boundaries, based on the target sequence uploaded. According to the mathematical algorithm used, each software provides a score rating the strength of the splice signal identified. Usually, a higher score corresponds to a stronger splice site. The following tools were used to test the functional impact of TP53 indel:

## SpliceView (http://bioinfo.itb.cnr.it/~webgene/wwwspliceview.html) is based on the basic Position Weight Matrix (PMW) model proposed by Shapiro and Senapathy with the advantage of considering mutual dependency between nucleotides in different positions.

## GENSCAN (http://hollywood.mit.edu/GENSCAN.html) exploits the Maximal Dependence Decomposition (MDD) model and provides a probability score describing the likelihood of identifying an exon-intron junction in the sequence provided by the user.

## NetGene2 (<http://www.cbs.dtu.dk/services/NetGene2/>) is based on an artificial Neural Network (NN) which is aimed at distinguishing true splice sites from blunders. This algorithm assigns a confidence score for each splice site position.

## NNSplice 0.9 (https://www.fruitfly.org/seq_tools/splice.html) is based on an artificial Neural Network (NN) as well but it generates a score to rank each splice site position.

## MaxEntScan (http://hollywood.mit.edu/burgelab/maxent/Xmaxentscan_scoreseq.html) is based on the Maximum Entropy Distribution (MED) model and provides a log-odd ratio to the 5’ and the 3’ splice site. As recommended by Houdayer *et al.*, the score of the mutant splice site should be at least 20% lower than the score of the corresponding wild-type splice site to be considered deleterious on splicing process.

## Human Splicing Finder (HSF, http://www.umd.be/HSF/HSF.shtml) incorporates both the PMW and the MED modelsto identify canonical splice sites, as well ESEs and ESSs.

## LC/MS-MS: Imatinib was quantified after a simple protein precipitation with methanol as extraction method. The analyte was separated on a Synergi Fusion RP C18 chromatographic column 4 μm, 50 x 2.0 mm coupled with a C18 precolumn (Phenomenex). Elution chromatography was carried out in gradient mode. The mass spectrometer was equipped with an electrospray ionization interface and operated in positive ion mode. The biological sample was analysed in Selected Reaction Monitoring mode following three different transitions. The quantification was performed using the 494.4 > 394.2 imatinib transition and employing imatinib-D8 as internal standard. The concentrations of the calibration curve (30-7500 ng/mL) cover those we expected to find in patients’ plasma. Finally, the developed method was validated according to the FDA and EMA guidelines on bioanalytical method validation assessing linearity, recovery, limit of detection, limit of quantification, matrix effect, inter- and intra-day precision and accuracy, selectivity, stability and reproducibility.

**Supplemetary Table S1** Human Actionable Solid Tumor Panel (Qiagen, Hilden, Germany). Regions covered.

| Exons | Hotspots | Whole Coding Region |
| --- | --- | --- |
| KIT | AKT1 | ERBB2 |
| PDGFRA | ALK | PIK3CA |
| EGFR | CTNNB1 | TP53 |
| KRAS | ERBB3 |  |
| NRAS | ESR1 |  |
| BRAF | FOXL2 |  |
|  | GNA11 |  |
|  | GNAQ |  |
|  | IDH1 |  |
|  | IDH2 |  |
|  | MET |  |
|  | RAF1 |  |
|  | RET |  |
